# Restraint Stress in Mice Alters Set of 25 miRNAs Which Regulate Stress- and Depression-Related mRNAs

**DOI:** 10.3390/ijms21249469

**Published:** 2020-12-12

**Authors:** Joanna Solich, Maciej Kuśmider, Agata Faron-Górecka, Paulina Pabian, Marta Dziedzicka-Wasylewska

**Affiliations:** Maj Institute of Pharmacology Polish Academy of Sciences, Smętna Street 12, 31-343 Kraków, Poland; kusmider@if-pan.krakow.pl (M.K.); gorecka@if-pan.krakow.pl (A.F.-G.); palach@if-pan.krakow.pl (P.P.); marta.dziedzicka-wasylewska@uj.edu.pl (M.D.-W.)

**Keywords:** *NET-KO* mice, *SWR/J* mice, microRNA, stress, depression, machine learning algorithm

## Abstract

In the present study, we aim to identify the effect of restrain stress (RS) on the expression of miRNAs in mouse serum. We used three genotypes of animals (mice with knock-out of the gene-encoding norepinephrine transporter, *NET-KO*; *C57BL/6J*, and *SWR/J*) which had previously been shown to display different sensitivity to RS, and focused on miRNAs which were altered by RS in the serum of all three genotypes. An analysis of miRNAs expression allowed for the identification of a set of 25 differentially expressed miRNAs; 10 were down-regulated compared to an appropriate control group of animals, while 15 were up-regulated. The application of DIANA-miRPath v. 3.0 allowed for the identification of selected pathways (KEGG) and Gene Ontology (GO) categories that were significantly controlled by these miRNAs, while miRWalk v. 3.0—the platform that used the machine learning based algorithm, TaRPmiR—was used to find their targets. The results indicate that 25 miRNAs, identified as altered upon RS in three genotypes of mice, are responsible for regulation of mRNA-encoding proteins that are key for the main hypotheses of depression; therefore, they may help to understand the link between stress and depression at the molecular level.

## 1. Introduction

Stress is often defined as a condition that seriously perturbs the psychological and physiological balance of an individual. Behavioural studies in rodents have demonstrated that environmental manipulations at different stages of life may have profound and lasting consequences. Such behavioural models include prenatal and postnatal stress [1] or environmental manipulations in adulthood [2], daily corticosterone administration or repeated physical restraint [3].

Both human and animal studies have shown that coping strategies are essential to minimize the impact of stress [4,5]; however, the underlying mechanisms of these responses have not been fully cognized, although they are known to depend on a combination of genetic and non-genetic factors that interact in complex ways. Since the role of stress has been repeatedly implicated as a causative factor of neuropsychiatric disorders including depression, studies of coping strategies have not lost their importance as they may help to understand the mechanisms of stress vulnerability vs. stress resilience. In our recent study, we used three strains of mice with various susceptibilities to stress: mice with a knocked-out gene encoding the norepinephrine transporter (*NET-KO* displaying a stress-resistant phenotype), as well as two strains of mice displaying two different stress-coping strategies, i.e., *C57BL/6J* (*WT*; wild type), and *SWR/J*. The procedure of restraint stress (RS, 4 h) was applied, and behavioural experiments (the forced-swim test and the sucrose-preference test) indicated that *NET-KO* and *SWR/J* mice were less sensitive to RS than WT mice [6].

Recently, many studies have focused on the idea that microRNAs (miRNAs) may act as mediators of the brain genomic response to stress [7,8,9,10,11]. Various miRNAs have been shown to be regulated by different kinds of stress and they are regarded as endogenous “hubs” (as Issler and Chen have put it) for the fine tuning of target gene expression or for “expression switch”, and may provide deeper insight into complex biological processes underlying the stress response [12].

The aim of the present study is to identify the expression of miRNAs upon RS in the serum of all three genotypes, regardless of their behavioural response to stress. Based on our previous studies [6] which allowed for identification of miRNAs (out of 768 miRNAs present on the TaqMan Array Rodent MicroRNA A+B Cards Set v3.0; Life Technologies) expressed in the serum of three genotypes (*WT*, *NET-KO* and *SWR/J*), we designed Custom TaqMan Array microRNA Cards (Life Technologies) with 191 miRNAs in order to study their expression upon RS in the serum of experimental animals.

## 2. Results

### 2.1. Analysis of miRNAs Expression

**Figure 1:**
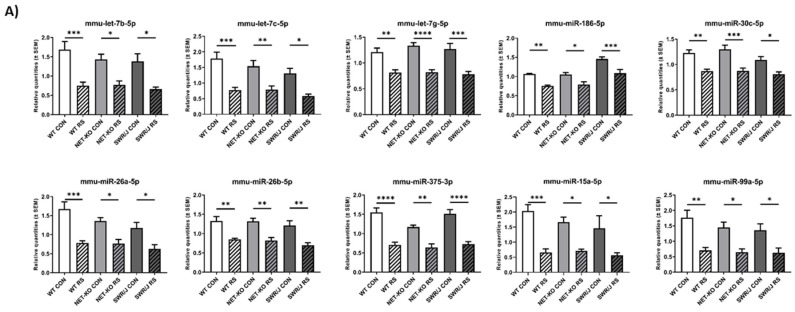

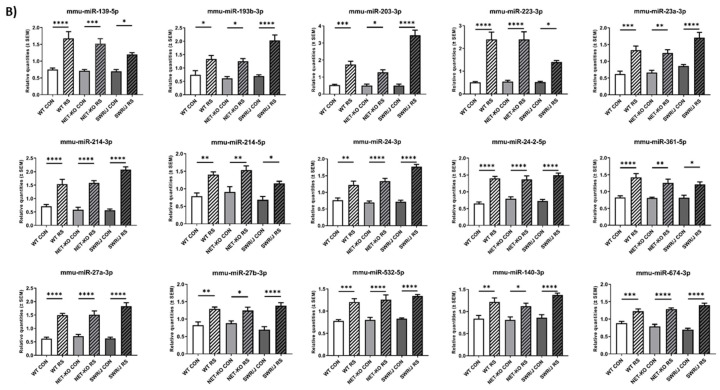
The level of miRNAs in the serum of three genotypes (WT, NET-KO, SWR/J) of mice under control (CON) and stress (RS) conditions. (**A**) Panel A contains all miRNAs downregulated after RS, while (**B**) panel B—miRNAs upregulated following RS. The significant changes after stress were marked on the graph: **** *p* < 0.0001; *** *p* ≤ 0.001; ** *p* ≤ 0.01; * *p* ≤ 0.05; *n* = 7. The remaining significant statistical differences are not marked on the graphs (for clarity reasons) but are listed in the Supplementary Materials (Table S4).

The 25 miRNAs were differently expressed in all genotypes after stress (Figure 1; normalised data—Table S2). The expression of group of miRNAs was reduced after RS as compared to control groups (Figure 1A). On the other hand, the expression of remaining miRNAs was enhanced upon RS, as compared to the controls (Figure 1B). The effect of RS on the miRNAs expression alterations was significant (Table S3A). For most of the miRNAs, the genotype effect was not significant; however, it was significant for 10 of them (Table S3B).

### 2.2. KEGG Pathway Analysis

**Figure 2:**
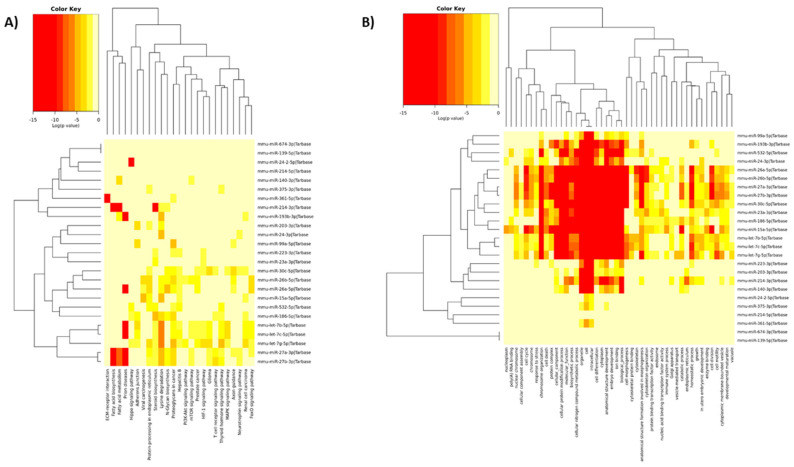
The relationship of miRNAs with Kyoto Encyclopedia of Genes and Genomes (KEGG) pathways (**A**) or Gene Ontology (GO) annotations (**B**). The heatmaps present links of miRNAs (placed on the right side) with KEGG pathways or GO annotations (placed at the bottom of the heatmaps). The miRNAs clusters were marked on the left side of the heatmaps, while KEGG pathway or GO annotation clusters—at the top of the heatmaps. The significance of linking miRNAs to KEGG pathways or GO annotations is color-coded (the scale in the upper, left corner).

The 25 miRNAs expressed differently upon RS in all three genotypes were analyzed to determine the processes in which they may be involved. As the result of this analysis, a heatmap presenting the relationship between miRNAs and KEGG pathways was created (Figure 2A). The miRNAs were clustered into four groups. Two of them showed relationships to most of indicated pathways. These groups contain the following miRNAs: mmu-miR-27a-3p, mmu-miR-27b-3p, mmu-let-7g-5p, mmu-let-7c-5p, mmu-let-7b-5p, mmu-miR-186-5p, mmu-miR-532-5p, mmu-miR-15a-5p, mmu-miR-26a-5p, mmu-miR-26b-5p and mmu-miR-30c-5p. In other clusters, single miRNAs also showed a significant link to KEGG pathways on the heatmap (mmu-miR-193b-3p, mmu-miR-214-3p, mmu-miR-361-5p and mmu-miR-24-2-5p).

The miRNAs described above were associated with 25 various KEGG pathways. The most interesting of these signaling pathways were the following: steroid biosynthesis, and signalling pathways of neurotrophin, thyroid hormone, and T-cell receptor as well as fatty acid biosynthesis and metabolism. Additionally, some KEGG pathways indicated by analysis were associated with second-messengers and signal transduction, such as MAPK, PI3K-Akt or mTOR signaling pathways.

### 2.3. GO Analysis

Gene ontology (GO) analysis included three aspects: molecular function, cellular component and biological process. The result of this analysis was indication of 46 annotations (Figure 2B). The miRNAs differentiating all three genotypes subjected to RS from control groups formed five clusters; one of them was associated with all annotations. This cluster included the following miRNAs: mmu-let-7g-5p, mmu-let-7c-5p, mmu-let-7b-5p, mmu-miR-15a-5p, mmu-miR-186-5p, mmu-miR-23a-3p, mmu-miR-30c-5p, mmu-miR-27a-3p, mmu-miR-27b-3p, mmu-miR-26a-5p and mmu-miR-26b-5p.

Two other clusters contained miRNAs (mmu-miR-140-3p, mmu-miR-214-3p, mmu-miR-203-3p, mmu-miR223-3p; mmu-miR-24-3p, mmu-miR-532-5p, mmu-miR-193b-3p, mmu-miR-99a-5p) significantly associated with some annotations, including chromosome organization, a cellular protein modification process, intracellular, cytoplasm, ion binding and homeostatic processes. Other interesting annotations were the following: protein binding transcription factor activity, nucleic acid binding transcription factor activity, immune system processes and, the most interesting—response to stress. The miRNAs associated with this last annotation were also associated with KEGG pathways, such as steroid biosynthesis or neurotrophin signaling pathway.

### 2.4. Linking miRNAs to Target Genes

**Figure 3:**
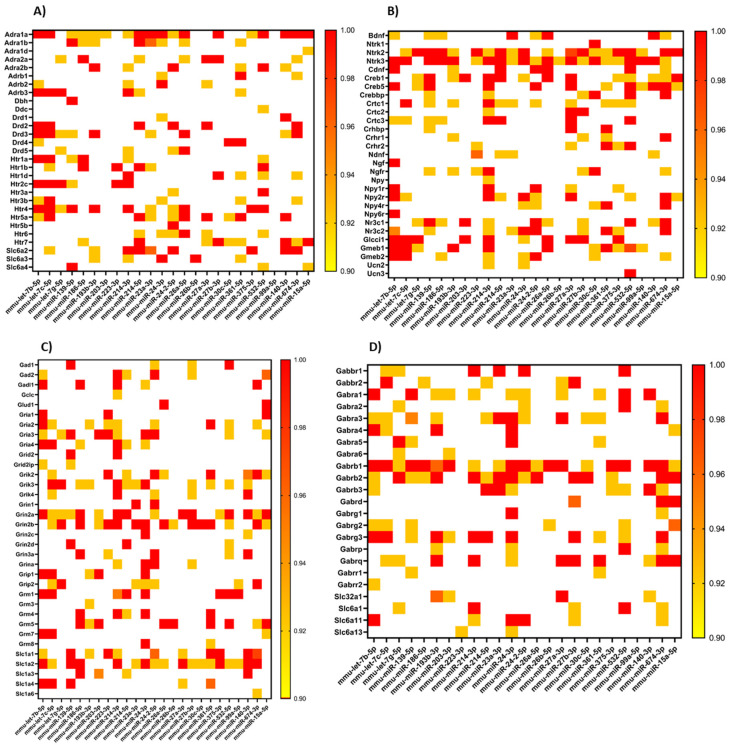

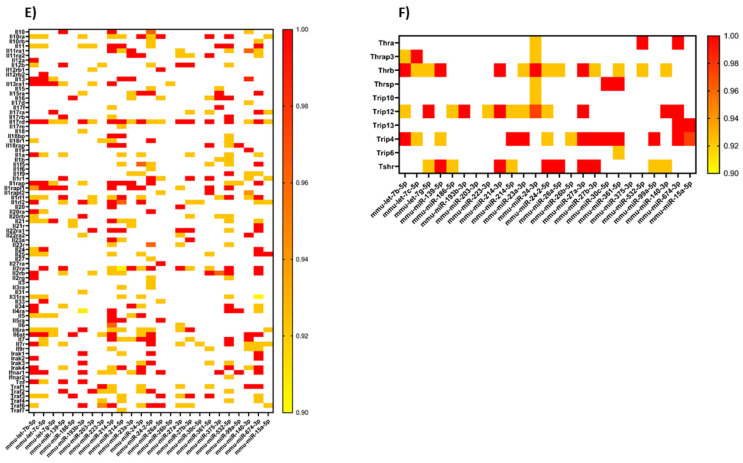
The mRNAs regulated by miRNAs, changed after stress in the serum of three genotypes (WT, NET-KO and SWR/J) of mice. The mRNAs were divided into six groups: receptors of monoamines and their transporters (**A**), neurotrophic factors, neuropeptides and their receptors together with glucocorticoid receptors (**B**), glutamate receptors and transporters (**C**), gamma-aminobutyric acid (GABA) receptors and transporters (**D**), interleukins and tumor necrosis factor together with the receptors as well as interferon receptors (**E**), thyroid hormone receptors (**F**). The relationship between mRNAs (placed on the left side) and the regulating miRNAs (placed at the bottom) are presented as heatmaps. The strength of probability of miRNA–mRNA interaction is shown as the *p* value and is color-coded (the scales presented on the right side of the heatmaps). The highest *p* value was indicated in red.

To find the target mRNAs for the miRNAs indicated by above analyses, the miRWalk platform was employed. This analysis indicated that miRNAs under study are involved in one way or another with regulation of 16,573 different mRNAs. Many miRNAs had the same mRNA target, which allowed for some groups of regulated mRNAs to be presented as the heatmaps (Figure 3). The strength of probability of miRNA–mRNA interaction was shown as the *p* value on the heatmap. The higher *p* value indicated the greater probability of specific mRNA regulation by miRNA. The highest *p* value was equal to 1, while the lowest one—selected during analysis—was equal to 0.9. Additionally, the results of the analysis are available in Supplementary Materials (Table S5). The selected mRNAs were divided into four groups, encoding the following: receptors of some monoamines and their transporters (Figure 3A), neurotrophic factors, neuropeptides and their receptors together with glucocorticoid receptors (Figure 3B), glutamate receptors and transporters (Figure 3C), gamma-aminobutyric acid (GABA) receptors and transporters (Figure 3D). In detail, these groups contain adrenergic, serotonin and dopamine receptors and transporters, the brain-derived neurotrophic factor (*Bdnf*), cerebral dopamine neurotrophic factor (*Cdnf*), neuron-derived neurotrophic factor (*Ndnf*), and nerve growth factor (*Ngf*) appeared among the neurotrophins, together with cyclic AMP response element binding-protein (*Creb*)—mediator of neurotrophin response. In turn, mRNA encoding neuropeptides such as neuropeptide Y (*Npy*), corticotropin-releasing hormone (*Crh*) receptors and urocortins (*Ucn*) are also regulated by the identified miRNAs as well as the mRNA encoding glucocorticoid (*Nr3c1*) and mineralocorticoid (*Nr3c2*) receptors. Additionally, ionotropic and metabotropic glutamate receptors could be found in the group in Figure 3C. Additionally, the mRNA-encoding enzymes involved in the synthesis and catabolism of glutamate as well as catalyzing synthesis and conversion of dopamine were also found to be regulated by miRNAs altered by RS in all three genotypes of mice, and were placed on the heatmaps.

Additionally, the two other interesting groups were identified. One of them contains interleukins and tumor necrosis factor together with the receptors as well as interferon receptors (Figure 3E). The mRNA-encoding interleukin-1 (*Il1*) and interleukin-6 (*Il6*) as well as their receptors can be found on this heatmap and kinases associated with the Il-1 signaling pathway were also in this group. The other group contains thyroid hormone receptors (Figure 3F), since the analysis indicated that the mRNA encoding thyroid stimulating hormone (*Tsh*) receptor can be regulated by the identified miRNAs.

### 2.5. Verification of the mRNAs Expression Indicated by In Silico Analysis

**Figure 4:**
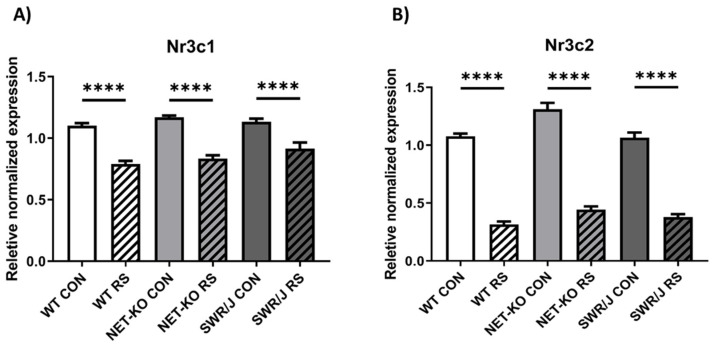
The expression of mRNA encoding the genes regulated by tested miRNAs in the liver of three genotypes (WT, NET-KO, SWR/J) of mice under the control condition (CON) and following stress (RS). Nuclear receptor subfamily 3, group C, member 1; glucocorticoid receptors (Nr3c1; (**A**)); nuclear receptor subfamily 3, group C, member 2; mineralocorticoid receptors (Nr3c2; (**B**)). **** *p* < 0.0001; *n* = 4. The significant statistical differences not marked on the graphs: A) WT CON vs. NET-KO RS *p* < 0.0001; WT CON vs. SWR/J RS *p* ≤ 0.001; NET-KO CON vs. WT RS *p* < 0.0001; NET-KO CON vs. SWR/J RS *p* < 0.0001; SWR/J CON vs. WT RS *p* < 0.0001; SWR/J CON vs. NET-KO RS *p* < 0.0001; WT RS vs. SWR/J RS *p* ≤ 0.05; B) WT CON vs. NET-KO CON *p* ≤ 0.001; WT CON vs. NET-KO RS *p* < 0.0001; WT CON vs. SWR/J RS *p* < 0.0001; NET-KO CON vs. SWR/J CON *p* ≤ 0.001; NET-KO CON vs. WT RS *p* < 0.0001; NET-KO CON vs. SWR/J RS *p* < 0.0001; SWR/J CON vs. WT RS *p* < 0.0001; SWR/J CON vs. NET-KO RS *p* < 0.0001.

To verify expression changes in mRNA encoding glucocorticoid (*Nr3c1*; Figure 4A; normalized data—Table S6) and mineralocorticoid (*Nr3c2*; Figure 4B; normalized data—Table S6) receptors, which were indicated by in silico analysis as regulated by miRNAs under investigation, the RT-qPCR reactions were performed in the mouse livers.

The expression of both examined mRNAs was reduced after stress (RS) in all tested genotypes. The effect of stress on mRNA expression was significant (*Nr3c1* (F(1,42) = 147.2; *p* < 0.0001; *Nr3c2* (F(1,42) = 716.6; *p* < 0.0001). Likewise, the effect of genotype on the changes of mRNA expression was significant in both cases (*Nr3c1* (F(2,42) = 3.893; *p* = 0.0281; *Nr3c2* (F(2,42) = 15.26; *p* < 0.0001).

## 3. Discussion

In the present study, we aimed to identify the effect of restrain stress (RS) on the expression of miRNAs in mouse serum. We used three genotypes of animals which had previously been shown to display different sensitivity to RS [6]; however, in the present study, we focused on miRNAs which were altered by stress in the serum of all three genotypes regardless of their behavioural response. An analysis of miRNAs expression allowed for the identification of a set of 25 miRNAs which were differently expressed; 10 were down-regulated as compared to an appropriate control group of animals, while 15 were up-regulated. Some of them were previously associated with stress and depression. Upon chronic social defeat stress, mmu-miR-24-2-5p, mmu-miR-27a-3p and mmu-miR-532-5p were altered in the rat serum, while maternal separation combined with unpredictable maternal stress changed the level of mmu-miR-375-3p [13,14]. Likewise, changes in miRNAs (mmu-miR-30c-5p and mmu-miR-375-3p) were found in the sperm of stressed male mice, and, in line with the idea that epigenetic changes may affect offspring, in subsequent studies, they were shown to do so [15,16]. It has been also shown, that some miRNAs, identified in the present studies, were altered in the blood of depressed patients. Both Maffioletti et al. and Fan et al. have shown that the level of mmu-miR-24-3p, mmu-miR-140-3p and mmu-miR-26b-5p was altered in the blood of major depressive disorder patients [17,18]. Gururajan et al. pointed to mmu-let-7b-5p and mmu-let-7c-5p as biomarkers for major depression [19]. They determined a lower level of these miRNAs in patient blood of patients. Likewise, we detected reduced expression of these miRNAs in RS mice serum.

The identified miRNAs were analysed using the KEGG pathway approach, which showed their possible involvement in signalling pathways of the neurotrophin, thyroid hormone, and the T-cell receptor, as well as fatty acid biosynthesis and metabolism and steroid biosynthesis. Additionally, some KEGG pathways indicated by our analyses were associated with second-messengers and signal transduction, such as MAPK, PI3K-Akt or mTOR signalling pathways, which have been shown as dysfunctional in depression and under stress [20,21]. Some annotations revealed by the GO analysis were found to be in line with these findings.

However, the most interesting results were obtained when—using the miRWalk platform—we linked miRNAs to their target genes and found that many of these miRNAs had the same mRNA targets. It must be kept in mind that the false positive rate of the target prediction algorithms is relatively high, but it must be emphasized that the miRWalk platform is based on a TarPmiR (Target Prediction for miRNAs) algorithm, which is a machine learning based method. This enables a new approach to investigate the miRNA–mRNA interaction. The TarPmiR applies a random-forest-based approach to integrate six conventional features and seven new features to predict miRNA target sites (thus, it is based on 13 features). The new features were not used by other tools, such as miRanda, TargetScan, DIANA-microT-CDS or TargetMiner. Therefore, TarPmiR complements the available tools by predicting sites that could not have been predicted without this approach [22]. Furthermore, the miRWalk platform covers datasets of TargetScan v.7.1, miRDB v.5.0—other datasets predicting miRNA binding sites—and validates information from miRTarBase v.7.0. Furthermore, it was also important that the platform is updated every 6 months [23].

All the mRNAs identified with this analysis were interesting; all of them encoded well-known proteins which are frequently said to have important or even crucial roles in stress and depression mechanisms and in the action mechanism of antidepressant drugs. Thus, the targets of the miRNAs which were altered by RS in all three genotypes of mice regardless of their behavioural response to RS included mRNAs encoding receptors of monoamines and their transporters, neurotrophic factors, neuropeptides and their receptors, glucocorticoid receptors, glutamate receptors and transporters, gamma-aminobutyric acid (GABA) receptors and transporters. Additionally, there were mRNAs encoding interleukins and the tumour necrosis factor together with the receptors, interferon receptors as well as thyroid hormone receptors. Data obtained using in silico analyses were verified by RT-qPCR reactions in the mouse liver for two investigated mRNAs regulated by miRNAs, namely glucocorticoid (*Nr3c1*) and mineralocorticoid (*Nr3c2*) receptors. This organ was selected as it was shown that mRNA encoding glucocorticoid receptors decreased significantly in rat liver after scalding stress [24]. Additionally, Hsu et al. have shown that bipolar disorder patients develop liver disease more often [25].

All the mRNAs shown in our analysis to be regulated by miRNAs, which in our experiment are altered by RS, are frequently found in the literature dealing with effects of stress itself, the role of stress as a cause of mental disorders, especially major depression and the action mechanism of antidepressant drugs [5,26,27,28,29,30].

There are many studies exploring the role of particular miRNA in the context of stress and depression. An example is that of sophisticated studies indicating down-regulation of miR-132 (induced by chronic unpredicted mild stress only in the hippocampus) and miR-22, which possibly increases monoamine oxidase A (MAO-A) gene expression by targeting *MAOA* mRNA [31]. Additionally, over-expression of miR-142, miR-34a, and miR-34c may reduce *MAOA* gene expression by inhibiting *SIRT1* gene expression [31]. In a recent study, Bahi and Dreyer [32] have found that in the hippocampus of mice, over-expression of the lethal-7 (let-7d) miRNA targets the dopamine D3 receptors and significantly improves depressive behaviour. Baudry et al. [33] showed that the serotonin transporter is a target of miR-16, while Issler et al. [34] found that the serotonin transporter and the serotonin receptor-1a transcripts are both targets of miR-135. Additionally, miR-1202 has been shown to be a regulatory factor of the metabotropic *GRM4* gene, which is involved in dopaminergic, glutamatergic, GABAergic, and serotonergic neurotransmission, and this finding is important in the context of studies indicating that down-regulation of miR-1202 in the prefrontal cortex of subjects with depression is negatively correlated with *GRM4* expression [35]. It has also been shown that miR-355 has effects similar to those of miR-1202 [36]. Important results have been provided by Roy et al. [37], who found that miR-124-3p is involved in regulating the *Gria4* expression in the prefrontal cortex of rats, which plays a role in the depressive phenotype.

All the above mentioned studies are important, but they may be treated as similar to studies of candidate gene variants specific for depression, which are poorly replicated [38]. On the other hand, our approach which does not focus on particular miRNA but rather searches without any bias for simultaneous alterations in their set, as has been shown in this study, allows for more thorough analysis and conclusions. By identifying a set of 25 key miRNAs sensitive to stress which converge at common target mRNAs encoding most genes long implicated as crucial for stress response as well as depression, we provide a platform which may be taken into account when the molecular basis of the link between stress and depression is considered.

## 4. Materials and Methods

### 4.1. Animals

Heterozygous mice (*Slc6a2^+/−^*; *C57BL/6J* background), obtained from Dr M. Caron [39] were mated with each other. Homozygous *WT* and *NET-KO* (*Slc6a2^tm1Mca^/IFPAS*) males, ca. 4.5 month old, were used for further experiments. Genotypes were as previously described [6].

The Swiss mice (*SWR/J*) were obtained from the Jackson Laboratory (USA). Six-week old males and females were mated with each other in the IFPAS (Maj Institute of Pharmacology Polish Academy of Sciences). The 4.5 month old males were used for further experiments.

The mice were housed in groups. Animals had free access to food and water and were kept at a constant room temperature (24 °C) under a 12 h light/dark cycle. Animals were kept according to the decision of the Minister of Environment (no. 88/2014) and the Local Bioethic Commission (199/2017).

The animals were divided into six groups: three different genotypes (*WT*, *NET-KO* and *SWR/J*) were compared under control conditions and following restraint stress (RS).

### 4.2. Restraint Stress (RS)

The seven male mice per group were placed in well-ventilated polypropylene-tubes (diameter 28 mm and length 110 mm), located in their home cage, for 4 h. During the immobilization phase, the mice did not have access to food and water. Next, they were left undisturbed in their home cage for 10 min.

### 4.3. Blood Collection

The trunk blood was collected from mice (RS and control group) of each genotype. Blood was left at room temperature for 20–30 min to clot. Then, it was centrifuged at 3000 rpm for 15 min at 4 °C and 3000 rpm for 5 min at 4 °C. The hemolysis in serum was determined by the absorbance of hemoglobin at 414 nm (A414) using NanoDrop ND-1000 (Thermo Fisher Scientific, Waltham, MA, USA). Serum samples with absorbance ≤0.3 were considered as unhemolyzed [40], and stored at −80 °C until further purification (Table S7 shows A414 for each sample).

### 4.4. Isolation of miRNAs from Serum

Total RNAs including small RNAs was isolated from 80 µL of the serum using an miRNeasy Serum/Plasma Kit (Qiagen, Germantown, MD, USA) according to the manufacturer’s instructions. The synthetic spiked-in ath-miR-159a was added to each sample at an amount of 3 × 10^9^ copies to monitor RNA extraction [41]. We used MS2 RNA (1 µg) as a carrier to increase recovery. The 400 µL of QIAzol lysis reagent and 80 µL of chloroform were added to each sample. The recovered amount of aqueous phase was always 240 µL. The 360 µL of 100% ethanol was added per sample. The quality and quantity of the isolated total RNA were evaluated by NanoDrop ND-1000 (Thermo Fisher Scientific, Waltham, MA, USA) and Experion microcapillary electrophoresis system (Bio-Rad, Hercules, CA, USA).

### 4.5. miRNA RT-qPCR Array

We designed Custom TaqMan Array MicroRNA Cards—192 format (Thermo Fisher Scientific, Waltham, MA, USA) based on our previous experiments [6]. One Custom TaqMan Array MicroRNA Card was used per sample. The samples were run in duplicates. The cDNA was synthetized by TaqMan MicroRNA Reverse Transcription Kit (Thermo Fisher Scientific, Waltham, MA, USA) with TaqMan Custom RT Pool according to the manufacturer’s instructions. The obtained cDNA was preamplified to increase the quantity of desired cDNA using TaqMan PreAmp Master Mix (Thermo Fisher Scientific, Waltham, MA, USA) with TaqMan Custom PreAmp Primers. The RT-qPCR reactions were performed with TaqMan Universal PCR Master Mix, No AmpErase UNG (Thermo Fisher Scientific, Waltham, MA, USA). The qPCRs were run on a QuantStudio 12K Flex System (Applied Biosystems, Foster City, CA, USA).

Data were further analyzed with QuantStudio 12K Flex Software (Applied Biosystems, Foster City, CA, USA). A Ct value above 30 was considered as undetectable miRNAs due to the preamplification. The same threshold equal to 0.2 was set for all samples for comparison. Then, the data were analyzed with qBasePLUS 3.1 software (Biogazelle, Gent, Belgium). The miRNAs: mmu-miR-301a-3p and mmu-miR-7a-1-3p—suitable for normalization—were generated by geNorm algorithm [42,43]. Afterwards, statistical analysis was carried out with GraphPad Prism 7.04 by two-way ANOVA to compare expression changes in each of the miRNAs between genotypes under control conditions and following RS. The Tukey’s post-test was used for multiple comparisons between groups. A value of *p* ≤ 0.05 was considered to be significant.

### 4.6. Identification of miRNA Targets In Silico

The miRNAs with expression levels significantly differentiating control from the RS group of each genotypes were subjected to further bioinformatics analyses.

The DIANA-miRPath v.3.0, an online software, was used for the assessment of miRNA regulatory roles and the identification of controlled pathways, as well as functional annotation [44]. Following pathway enrichment analysis using Fisher’s Exact Test (hypergeometric distribution), the miRNAs targets were selected from TarBase v7.0. A value of *p* ≤ 0.05 was considered to be significant and FDR correction was selected.

Additionally, the miRWalk v.3.0 was used to find target genes regulated by identified miRNAs [23]. This is the platform that used a machine learning based algorithm—TarPmiR—to predict miRNA-target interaction [22]. Otherwise, the miRWalk framework covers datasets of TargetScan v.7.1, miRDB v.5.0 and validated information from miRTarBase v.7.0 [45,46,47]. The appropriate species, 3′UTR region and *p* value equal to 0.9 were selected from the degree filter during analysis. Then, the selected results of the computational approach were validated experimentally at the mRNA level.

### 4.7. Isolation of mRNAs from the Liver

The livers of four mice of each genotype under control and stress conditions were separated 10 min after RS, frozen and stored at −80 °C until further use. The TRI Reagent (Sigma-Aldrich, St. Louis, MO, USA) was used for RNA purification, according to the manufacturer’s instruction. The livers were homogenized with 1 mL of reagent per sample, in the homogenizer stirrer (Glas-Col, Terre Haute, IN, USA). The amount of aqueous phase was always 400 µL. The quality and quantity of the isolated total RNA were evaluated as described above (Section 4.4). Samples that passed the quality threshold (RIN > 8.0) were used for further experiments.

### 4.8. Quantitative RT-qPCR Analyses of Individual mRNAs

The RNA was reverse-transcribed to cDNA transcripts according to the manufacturer’s protocol, using High Capacity cDNA Reverse Transcription Kit (Thermo Fisher Scientific, Waltham, MA, USA). For RT-qPCR reactions, TaqMan Universal Master Mix II (no UNG) and TaqMan Gene Expression Assay (Thermo Fisher Scientific, Waltham, MA, USA) were added to 20 ng of the cDNA template. The list of TaqMan Gene Expression Assays was included in the Supplementary Materials (Table S1). The real-time PCR reactions were carried out in duplicates using CFX96 Touch Real-Time PCR Detection System (Bio-Rad, Hercules, CA, USA) according to the manufacturer’s instructions. The results were normalized to β-actin (Actb) and glyceraldehyde-3-phosphate dehydrogenase (Gapdh). Then, the results were analyzed with CFX Manager Software (Version 3.1; Bio-Rad, Hercules, CA, USA) using the ∆∆Cq method to obtain the relative normalized expression values. GraphPad Prism (Version 7.04) was used for statistical analysis. Two-way ANOVA (followed by Tukey’s post-test) was used to determine the statistical significance of differences of genotype and stress response. The *p*-values ≤ 0.05 were considered statistically significant.

## Supplementary Materials

Supplementary materials can be found at https://www.mdpi.com/1422-0067/21/24/9469/s1. Table S1: The list of TaqMan Gene Expression Assays added to RT-qPCR reactions. Table S2: The normalized data of miRNA RT-qPCR Arrays. Table S3A: The results of statistical analysis of the effect of RS on the miRNAs expression in the serum. The analysis (two-way ANOVA) was carried out using GraphPad Prism 7.04. Table S3B: The results of statistical analysis of the effect of the genotype on the miRNAs expression in the serum. The analysis (two-way ANOVA) was carried out using GraphPad Prism 7.04. Table S4: The significant statistical differences, not marked on the graphs at the Figure 1. Table S5: The results of miRWalk analysis for selected mRNAs. Table S6: The normalized data of mRNA RT-qPCR. Table S7: The absorbance at 414 nm for all samples.
