# Supplementary material for: Restraint Stress in Mice Alters Set of 25 miRNAs Which Regulate Stress- and Depression-Related mRNAs
Source: Int J Mol Sci. 2020 Dec 12;21(24):9469. doi: 10.3390/ijms21249469 (PMC7763317; doi:10.3390/ijms21249469)
Supplement: Supplementary file 1 [file ijms-21-09469-s001.zip › Table S1.docx]

Table S1

| **Target name** | **Gene** | **Assay ID** |
| --- | --- | --- |
| nuclear receptor subfamily 3, group C, member 1; glucocorticoid receptor; GR | Nr3c1 | Mm00433832_m1 |
| nuclear receptor subfamily 3, group C, member 2; mineralocorticoid receptor; MR | Nr3c2 | Mm01241596_m1 |
| glyceraldehyde-3-phosphate dehydrogenase | Gapdh | Mm99999915_g1 |
| actin, beta | Actb | Mm00607939_s1 |
|  |  |  |
|  |  |  |
