# Supplementary material for: Restraint Stress in Mice Alters Set of 25 miRNAs Which Regulate Stress- and Depression-Related mRNAs
Source: Int J Mol Sci. 2020 Dec 12;21(24):9469. doi: 10.3390/ijms21249469 (PMC7763317; doi:10.3390/ijms21249469)
Supplement: Supplementary file 1 [file ijms-21-09469-s001.zip › Table S3.docx]

**Table S3.A**

The results of statistical analysis of the effect of RS on the miRNAs expression in the serum. The analysis (two-way ANOVA) was carried out using GraphPad Prism 7.04.

| **microRNAs** | **F(DFn, DFd); P value** |
| --- | --- |
| mmu-let-7b-5p | F(1,36) = 44.21; p < 0.0001 |
| mmu-let-7c-5p | F(1,36) = 49.48; p < 0.0001 |
| mmu-let-7g-5p | F(1,36) = 67.04; p < 0.0001 |
| mmu-miR-186-5p | F(1,36) = 44.07; p < 0.0001 |
| mmu-miR-26a-5p | F(1,34) = 44.27; p < 0.0001 |
| mmu-miR-26b-5p | F(1,35) = 47.61; p < 0.0001 |
| mmu-miR-30c-5p | F(1,36) = 51.53; p < 0.0001 |
| mmu-miR-375-3p | F(1,34) = 105.2; p < 0.0001 |
| mmu-miR-99a-5p | F(1,33) = 39.28; p < 0.0001 |
| mmu-miR-15a-5p | F(1,30) = 39.12; p < 0.0001 |
| mmu-miR-139-5p | F(1,36) = 67.24; p < 0.0001 |
| mmu-miR-193b-3p | F(1,35) = 65.86; p < 0.0001 |
| mmu-miR-203-3p | F(1,35) = 139.3; p < 0.0001 |
| mmu-miR-223-3p | F(1,36) = 95.53; p < 0.0001 |
| mmu-miR-214-3p | F(1,36) = 176.3; p < 0.0001 |
| mmu-miR-214-5p | F(1,36) = 45.15; p < 0.0001 |
| mmu-miR-24-3p | F(1,36) = 141.6; p < 0.0001 |
| mmu-miR-24-2-5p | F(1,36) = 164.0; p < 0.0001 |
| mmu-miR-27a-3p | F(1,36) = 162.4; p < 0.0001 |
| mmu-miR-27b-3p | F(1,36) = 57.20; p < 0.0001 |
| mmu-miR-23a-3p | F(1,35) = 72.07; p < 0.0001 |
| mmu-miR-361-5p | F(1,35) = 52.76; p < 0.0001 |
| mmu-miR-532-5p | F(1,36) = 86.93; p < 0.0001 |
| mmu-miR-140-3p | F(1,36) = 49.82; p < 0.0001 |
| mmu-miR-674-3p | F(1,36) = 134.6; p < 0.0001 |

**Table S3.B**

The results of statistical analysis of the effect of the genotype on the miRNAs expression in the serum. The analysis (two-way ANOVA) was carried out using GraphPad Prism 7.04.

| **microRNAs** | **F(DFn, DFd); P value** |
| --- | --- |
| mmu-miR-186-5p | F(2,36) = 25.22; p < 0.0001 |
| mmu-miR-26a-5p | F(2,34) = 3.635; p = 0.0371 |
| mmu-miR-375-3p | F(2,34) = 4.244; p = 0.0226 |
| mmu-miR-193b-3p | F(2,35) = 6.141; p = 0.0052 |
| mmu-miR-203-3p | F(2,35) = 22.69; p < 0.0001 |
| mmu-miR-223-3p | F(2,36) = 4.60; p = 0.0166 |
| mmu-miR-214-5p | F(2,36) = 4.315; p = 0.0209 |
| mmu-miR-24-3p | F(2,36) = 7.051; p = 0.0026 |
| mmu-miR-23a-3p | F(2,35) = 6.417; p = 0.0042 |
| mmu-miR-140-3p | F(2,36) = 4.675; p = 0.0157 |
