# Supplementary material for: Restraint Stress in Mice Alters Set of 25 miRNAs Which Regulate Stress- and Depression-Related mRNAs
Source: Int J Mol Sci. 2020 Dec 12;21(24):9469. doi: 10.3390/ijms21249469 (PMC7763317; doi:10.3390/ijms21249469)
Supplement: Supplementary file 1 [file ijms-21-09469-s001.zip › Table S4.docx]

| **microRNAs** | **significant statistical differences** |
| --- | --- |
| mmu-let-7b-5p | WT CON vs. NET-KO RS p ≤ 0.001; WT CON vs. SWR/J RS p ≤ 0.001; NET-KO CON vs. WT RS p ≤ 0.05; NET-KO CON vs. SWR/J RS p ≤ 0.01; SWR/J CON vs. WT RS p ≤ 0.05; SWR/J CON vs. NET-KO RS p ≤ 0.05 |
| mmu-let-7c-5p | WT CON vs. NET-KO RS p ≤ 0.001; WT CON vs. SWR/J RS p < 0.0001; NET-KO CON vs. WT RS p ≤ 0.01; NET-KO CON vs. SWR/J RS p ≤ 0.001 |
| mmu-let-7g-5p | WT CON vs. NET-KO RS p ≤ 0.01; WT CON vs. SWR/J RS p ≤ 0.01; NET-KO CON vs. WT RS p < 0.0001; NET-KO CON vs. SWR/J RS p < 0.0001; SWR/J CON vs. WT RS p ≤ 0.001; SWR/J CON vs. NET-KO RS p ≤ 0.001 |
| mmu-miR-186-5p | WT CON vs. SWR/J CON p ≤ 0.001; WT CON vs. NET-KO RS p ≤ 0.05; NET-KO CON vs. SWR/J CON p ≤ 0.001; NET-KO CON vs. WT RS p ≤ 0.05; SWR/J CON vs. WT RS p < 0.0001; SWR/J CON vs. NET-KO RS p < 0.0001; WT RS vs. SWR/J RS p ≤ 0.01; NET-KO RS vs. SWR/J RS p ≤ 0.05 |
| mmu-miR-30c-5p | WT CON vs. NET-KO RS p ≤ 0.01; WT CON vs. SWR/J RS p ≤ 0.001; NET-KO CON vs. WT RS p ≤ 0.001; NET-KO CON vs. SWR/J RS p < 0.0001 |
| mmu-miR-26a-5p | WT CON vs. NET-KO RS p ≤ 0.001; WT CON vs SWR/J RS p < 0.0001; NET-KO CON vs. WT RS p ≤ 0.05; NET-KO CON vs. SWR/J RS p ≤ 0.01 |
| mmu-miR-26b-5p | WT CON vs. NET-KO RS p ≤ 0.01; WT CON vs. SWR/J RS p ≤ 0.001; NET-KO CON vs WT RS p ≤ 0.01; NET-KO CON vs. SWR/J RS p ≤ 0.001; SWR/J CON vs. NET-KO RS p ≤ 0.05 |
| mmu-miR-375-3p | WT CON vs. NET-KO CON p ≤ 0.05; WT CON vs NET-KO RS p < 0.0001; WT CON vs. SWR/J RS p < 0.0001; NET-KO CON vs. WT RS p ≤ 0.01; NET-KO CON vs SWR/J RS p ≤ 0.01; SWR/J CON vs. WT RS p < 0.0001; SWR/J CON vs. NET-KO RS p < 0.0001 |
| mmu-miR-15a-5p | WT CON vs. NET-KO RS p ≤ 0.01; WT CON vs. SWR/J RS p ≤ 0.001; NE-KO CON vs. WT RS p ≤ 0.05; NET-KO CON vs. SWR/J RS p ≤ 0.05 |
| mmu-miR-99a-5p | WT CON vs. NET-KO RS p ≤ 0.01; WT CON vs. SWR/J RS p ≤ 0.001; NET-KO CON vs. WT RS p ≤ 0.05; NET-KO CON vs. SWR/J RS p ≤ 0.05 |
| mmu-miR-139-5p | WT CON vs. NET-KO RS p ≤ 0.001; NET-KO CON vs. WT RS p < 0.0001; NET-KO CON vs. SWR/J RS p ≤ 0.05; SWR/J CON vs. WT RS p < 0.0001; SWR/J CON vs. NET-KO RS p < 0.0001; WT RS vs. SWR/J RS p ≤ 0.05 |
| mmu-miR-193b-3p | WT CON vs SWR/J RS p < 0.0001; NET-KO CON vs. WT RS p ≤ 0.01; NET-KO CON vs. SWR/J RS p < 0.0001; SWR/J CON vs. WT RS p ≤ 0.05; SWR/J CON vs. NET-KO RS p ≤ 0.05; WT RS vs. SWR/J RS p ≤ 0.01; NET-KO RS vs. SWR/J RS p ≤ 0.01 |
| mmu-miR-203-3p | WT CON vs. NET-KO RS p ≤ 0.05; WT CON vs. SWR/J RS p < 0.0001; NET-KO CON vs. WT RS p ≤ 0.001; NET-KO CON vs. SWR/J RS p < 0.0001; SWR/J CON vs. WT RS p ≤ 0.001; SWR/J CON vs. NET-KO RS p ≤ 0.05; WT RS vs. SWR/J RS p < 0.0001; NET-KO RS vs. SWR/J RS p < 0.0001 |
| mmu-miR-223-3p | WT CON vs. NET-KO RS p < 0.0001; WT CON vs. SWR/J RS p ≤ 0.05; NET-KO CON vs. WT RS p < 0.0001; NET-KO CON vs. SWR/J RS p ≤ 0.05; SWR/J CON vs. WT RS p < 0.0001; SWR/J CON vs. NET-KO RS p < 0.0001; WT RS vs. SWR/J RS p ≤ 0.01; NET-KO RS vs. SWR/J RS p ≤ 0.01 |
| mmu-miR-214-3p | WT CON vs. NET-KO RS p < 0.0001; WT CON vs. SWR/J RS p < 0.0001; NET-KO CON vs. WT RS p < 0.0001; NET-KO CON vs. SWR/J RS p < 0.0001; SWR/J CON vs. WT RS p < 0.0001; SWR/J CON vs. NET-KO RS p < 0.0001; WT RS vs. SWR/J RS p ≤ 0.01; NET-KO RS vs. SWR/J RS p ≤ 0.05 |
| mmu-miR-214-5p | WT CON vs NET-KO RS p ≤ 0.001; NET-KO CON vs. WT RS p ≤ 0.05; SWR/J CON vs. WT RS p ≤ 0.001; SWR/J CON vs. NET-KO RS p < 0.0001 |
| mmu-miR-24-3p | WT CON vs. NET-KO RS p < 0.0001; WT CON vs. SWR/J RS p < 0.0001; NET-KO CON vs. WT RS p ≤ 0.001; NET-KO CON vs. SWR/J RS p < 0.0001; SWR/J CON vs. WT RS p ≤ 0.001; SWR/J CON vs. NET-KO RS p < 0.0001; WT RS vs. SWR/J RS p ≤ 0.001; NET-KO RS vs. SWR/J RS p ≤ 0.01 |
| mmu-miR-24-2-5p | WT CON vs. NET-KO RS p < 0.0001; WT CON vs. SWR/J RS p < 0.0001; NET-KO CON vs. WT RS p < 0.0001; NET-KO CON vs. SWR/J RS p < 0.0001; SWR/J CON vs. WT RS p < 0.0001; SWR/J CON vs. NET-KO RS p < 0.0001 |
| mmu-miR-27a-3p | WT CON vs. NET-KO RS p < 0.0001; WT CON vs. SWR/J RS p < 0.0001; NET-KO CON vs. WT RS p < 0.0001; NET-KO vs. SWR/J RS p < 0.0001; SWR/J CON vs. WT RS p < 0.0001; SWR/J CON vs. NET-KO RS p < 0.0001 |
| mmu-miR-27b-3p | WT CON vs. NET-KO RS p ≤ 0.05; WT CON vs SWR/J RS p ≤ 0.001; NET-KO CON vs. WT RS p ≤ 0.05; NET-KO CON vs. SWR/J RS p ≤ 0.01; SWR/J CON vs. WT RS p ≤ 0.001; SWR/J CON vs. NET-KO RS p ≤ 0.001 |
| mmu-miR-23a-3p | WT CON vs. NET-KO RS p ≤ 0.01; WT CON vs. SWR/J RS p < 0.0001; NET-KO CON vs. WT RS p ≤ 0.01; NET-KO CON vs. SWR/J RS p < 0.0001; SWR/J CON vs. WT RS p ≤ 0.05; NET-KO RS vs. SWR/J RS p ≤ 0.05 |
| mmu-miR-361-5p | WT CON vs NET-KO RS p ≤ 0.01; WT CON vs. SWR/J RS p ≤ 0.05; NET-KO CON vs. WT RS p < 0.0001; NET-KO CON vs. SWR/J RS p ≤ 0.05; SWR/J CON vs. WT RS p < 0.0001; SWR/J CON vs. NET-KO RS p ≤ 0.01 |
| mmu-miR-532-5p | WT CON vs. NET-KO RS p < 0.0001; WT CON vs. SWR/J RS p < 0.0001; NET-KO CON vs WT RS p ≤ 0.001; NET-KO CON vs. SWR/J RS p < 0.0001; SWR/J CON vs. WT RS p ≤ 0.01; SWR/J CON vs. NET-KO RS p ≤ 0.001 |
| mmu-miR-140-3p | WT CON vs. SWR/J RS p < 0.0001; NET-KO CON vs. WT RS p ≤ 0.01; NET-KO CON vs. SWR/J RS p < 0.0001; SWR/J CON vs. WT RS p ≤ 0.05 |
| mmu-miR-674-3p | WT CON vs. NET-KO RS p < 0.0001; WT CON vs. SWR/J RS p < 0.0001; NET-KO CON vs. WT RS p < 0.0001; NET-KO CON vs SWR/J RS p < 0.0001; SWR/J CON vs. WT RS p < 0.0001; SWR/J CON vs. NET-KO RS p < 0.0001 |
